# Human and porcine IgG subclasses differentially engage porcine Fc receptors

**DOI:** 10.1093/discim/kyag018

**Published:** 2026-09-21

**Authors:** Ashutosh Vats, Charlotte May, Elliot Moorhouse, Elena Lokhman, Sylvia Crossley, Abigail Hay, Marie Bonnet-Di Placido, Simon P Graham, Pramila Rijal, Alain Townsend, William Mwangi, John C Schwartz, Danish Munir, John A Hammond, Elma Tchilian, Basudev Paudyal

**Affiliations:** Host Response, The Pirbright Institute, Woking, UK; Host Response, The Pirbright Institute, Woking, UK; Host Response, The Pirbright Institute, Woking, UK; Host Response, The Pirbright Institute, Woking, UK; Host Response, The Pirbright Institute, Woking, UK; Host Response, The Pirbright Institute, Woking, UK; Host Response, The Pirbright Institute, Woking, UK; Host Response, The Pirbright Institute, Woking, UK; Chinese Academy of Medical Science Oxford Institute, Nuffield Department of Medicine, University of Oxford, Oxford, UK; MRC Translational Immune Discovery Unit, Weatherall Institute of Molecular Medicine, Radcliffe Department of Medicine, University of Oxford, Oxford, UK; Chinese Academy of Medical Science Oxford Institute, Nuffield Department of Medicine, University of Oxford, Oxford, UK; MRC Translational Immune Discovery Unit, Weatherall Institute of Molecular Medicine, Radcliffe Department of Medicine, University of Oxford, Oxford, UK; Host Response, The Pirbright Institute, Woking, UK; Host Response, The Pirbright Institute, Woking, UK; Host Response, The Pirbright Institute, Woking, UK; Host Response, The Pirbright Institute, Woking, UK; Host Response, The Pirbright Institute, Woking, UK; Host Response, The Pirbright Institute, Woking, UK

**Keywords:** porcine IgG subclasses, human IgG subclasses, porcine FcγRs, porcine FcRn, affinity Fc-FcγRs interactions, porcine Fc-mediated functions

## Abstract

Antibody fragment crystallizable (Fc)–mediated effector functions and pharmacokinetics are governed by interactions between immunoglobulin G (IgG) subclasses and Fc gamma receptors (FcγRs) and Fc neonatal receptor (FcRn). Although pigs are increasingly used as translational models for therapeutic antibody evaluation, the interactions between porcine Fc receptors (pFcγRs) and porcine (pIgG) or human IgG (hIgG) subclasses remain poorly defined. Here, we characterized the interactions of pIgG and hIgG subclasses with pFcγRs and pFcRn and assessed their ability to mediate Fc-dependent effector functions in porcine immune cells. Using biolayer interferometry, we demonstrated subclass-dependent differences in receptor binding, driven primarily by dissociation kinetics. Porcine IgG2 and pIgG5c exhibited stable FcγR interactions, whereas pIgG3 and pIgG5b showed weak binding to several low-affinity receptors (FcγRIIa, FcγRIIb, and FcγRIIIa). Human IgG subclasses displayed distinct binding profiles to pFcγRs, with hIgG3 showing the strongest and hIgG2 the weakest binding affinity. Furthermore, hIgG1 and hIgG4 did not bind to pFcγRIIIa. Functional studies demonstrated that hIgGs mediate Fc-dependent phagocytosis and natural killer cell degranulation, although receptor-binding affinity did not always correlate with functional activity. Comparative phylogenetic and functional analyses further revealed that FcγR-binding phenotypes are only partially predicted by Fc domain sequence similarity. All pIgG and hIgG subclasses interacted with pFcRn in a comparable pH-dependent manner. These findings demonstrate substantial, but incomplete, conservation of Fc biology between humans and pigs. While supporting the use of pigs as a translational model for antibody pharmacokinetic studies, they also indicate the need for integrated biophysical, structural, and functional analyses before extrapolating Fc-mediated therapeutic activity between species.

## Graphical Abstract

**Graphical Abstract:**
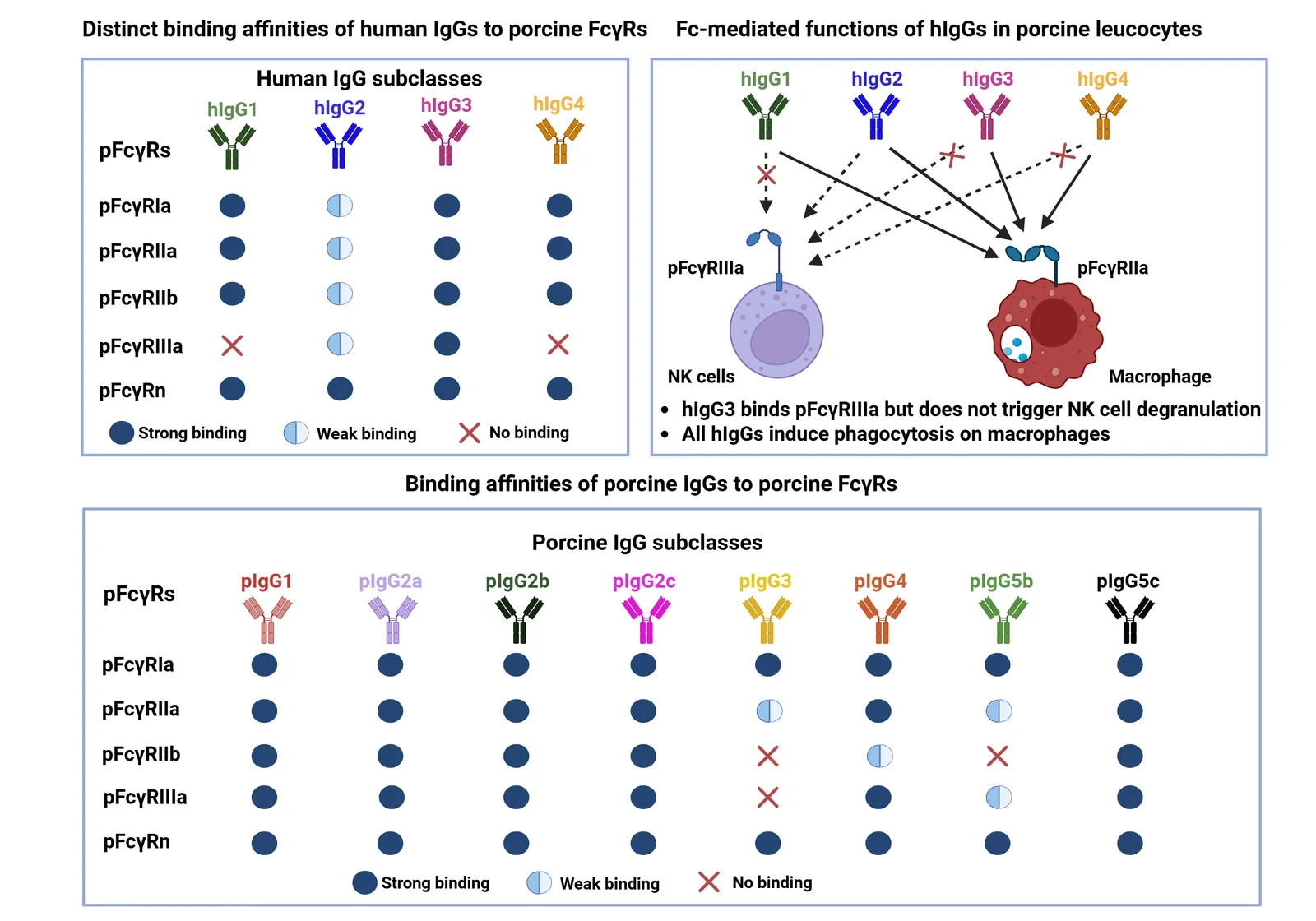

## Introduction

Antibodies mediate protection through antigen recognition and fragment crystallizable (Fc)–dependent recruitment of immune effector mechanisms. These functions are mediated by interactions between the Fc domain and Fc receptors (FcRs) expressed on immune cells, as well as by activation of the complement component C1q. FcRs are specific for each antibody isotypes: FcγRs for IgG, FcαRs for IgA, FcεRs for IgE, FcμRs for IgM, and FcδRs for IgD [1]. FcγRs are activated primarily by multivalent antigen bound antibody complexes rather than monomeric antibodies, leading to receptor clustering and downstream signalling that induces cellular effector responses such as antibody-dependent cellular cytotoxicity (ADCC), antibody-dependent cellular phagocytosis (ADCP), cytokine secretion, and antigen presentation [2].

In humans, FcγRs comprise the high-affinity receptor FcγRI (CD64) and the low-affinity receptors FcγRIIa (CD32A), FcγRIIb (CD32B), FcγRIIc (CD32C), FcγRIIIa (CD16A), and FcγRIIIb (CD16B) encoded by *FCGR1*, *FCGR2A*, *FCGR2B*, *FCGR2C*, *FCGR3A*, and *FCGR3B*. Activating FcγRs signal through FcR γ-chains containing immunoreceptor tyrosine-based activation motifs (ITAMs), whereas inhibitory receptors contain intrinsic immunoreceptor tyrosine-based inhibitory motifs (ITIMs) that dampen immune activation [3]. FcγRI is highly conserved in mammalian species and is predominantly expressed on monocytes, macrophages, and dendritic cells, where it mediates ADCP, antigen presentation, and cytokine production [4, 5]. In contrast, most other FcγRs are low-affinity receptors that require immune complex formation for efficient binding. FcγRIIa promotes ADCP, cytokine production, and platelet activation, while FcγRIIb negatively regulates signalling to maintain immune homeostasis. FcγRIII is expressed primarily on monocytes, macrophages, dendritic cells, and natural killer (NK) cells and plays an important role in cytotoxic effector responses [6].

Pigs are increasingly recognized as a high-fidelity model for biomedical research, including the evaluation of pharmacokinetics and efficacy of therapeutic monoclonal antibodies (mAbs) [7–11]. Their anatomical, physiological, immunological, and genetic similarities to humans make them particularly suitable for preclinical studies of human therapeutic mAbs. Despite the growing use of pigs in translational research, porcine (p) FcγRs remain less well characterized than their human and murine counterparts, although recent molecular and functional studies have begun to define their structure and biology [5]. In Göttingen minipigs, pFcγRIa, pFcγRIIa, and pFcγRIIb have been characterized and shown that FcγRIIa share substantial extracellular domain homology with human and bovine FcγRs [5, 12]. However, unlike human FcγRIIa, the porcine FcγRIIa lacks an intrinsic ITAM and signal through association with the FcγR chain indicating differences in signalling and regulatory functions to human [5]. These studies further demonstrated that hIgG1 did not bind pFcγRIIIa, suggesting that FcγRIIIa-dependent effector functions, particularly NK cell-mediated ADCC, may be underestimated when human therapeutic antibodies are evaluated in pigs [12].

Comparative genomic analyses have shown that pigs and cattle have orthologues of most human *FCGR* genes, except for *FCGR2C* and *FCGR3B* (Noble *et al*., 2023). Human *FCGR1A* is orthologous to murine and porcine *FCGRIA*, while human *FCGRIIA* is orthologous to receptors in pigs, non-human primates, cattle, and mice, although in mice the corresponding receptor is termed *FCGRIII* [5, 13]. Similarly, human *FCGRIIIA* is orthologous to porcine *FCGRIIIA*, whereas the murine orthologue is designated *FCGRIV* [14]. In pigs, pFcγRIIIa associates with a cathelin-related molecule, that may link innate and adaptive immune responses, suggesting species-specific porcine FcγR regulatory functions [15]. These differences in FcγR repertoire, structure, and nomenclature between species complicate direct comparisons and indicate the need for caution when translating Fc receptor biology between animal models and humans.

In addition to FcγRs, the neonatal Fc receptor (FcRn) plays a critical role in antibody biology by regulating IgG transport, tissue distribution, and serum half-life [16, 17]. Although FcRn function is conserved across mammals, species-specific differences in receptor structure, IgG binding affinity, and tissue distribution can significantly affect mAb half-life and therapeutic efficacy. For example, murine FcRn binds hIgG with unusually high affinity, whereas porcine FcRn appears to display pharmacokinetic characteristics more comparable to humans, supporting the use of pigs as a translational model for therapeutic mAb studies [9, 18].

We have previously characterized the Fc-mediated functions of pIgG subclasses and examined their binding to monocytes, monocyte-derived macrophages, and NK cells [19]. Using a similar approach, we also investigated the Fc-mediated functions of bovine IgG subclasses and the distribution of bovine FcγRs in leucocytes and analysed their interactions with recombinant bovine FcγRs [20]. Building on these studies, here we characterized the interactions between pIgG subclasses and pFcγRs. Given the increasing use of pigs as translational models for evaluation of human mAbs, we also assessed the binding and Fc-mediated functions of hIgG1, hIgG2, hIgG3, and hIgG4 subclasses with pFcγR. We further investigated interactions with porcine FcRn, a key determinant of antibody pharmacokinetics. Together, this study provides important insights into Fc receptor biology in pigs and discusses their use for the preclinical evaluation of therapeutic mAbs.

## Results

### Interactions and affinities of pIgG subclasses with pFcγRs

The porcine mAb pb27, which targets the K130 site of haemagglutinin (HA) of the pandemic H1N1 virus (H1N1pdm09), was isotype-switched into all pIgG subclasses, pIgG1, pIgG2a, pIgG2b, pIgG2c, pIgG3, pIgG4, pIgG5b, and pIgG5c, as previously described [21, 22]. Recombinant pFcγRs were expressed as soluble proteins comprising the extracellular domain fused to an Avi-tag and a C-terminal six-histidine tag. SDS-PAGE analysis showed that the proteins were of high purity with no detectable degradation products (Supplementary Figure S1a and b). Binding interactions between monomeric pIgG Fc regions and pFcγRs were analysed by biolayer interferometry (BLI) using streptavidin biosensors. Recombinant pFcγRs were immobilized onto the sensor surface, and binding kinetics were determined using a five-point, 2-fold serial dilution of IgG starting at 400 nM, as shown in representative sonograms in Fig. 1a. For most IgG-FcγR interactions, the dissociation phase showed a biphasic profile characterized by an initial rapid dissociation followed by a slower phase, suggesting stabilized binding interactions. To improve curve fitting and kinetic analysis, only the first 15 sec (s) of the dissociation phase were included in the calculations.

**Figure 1:**
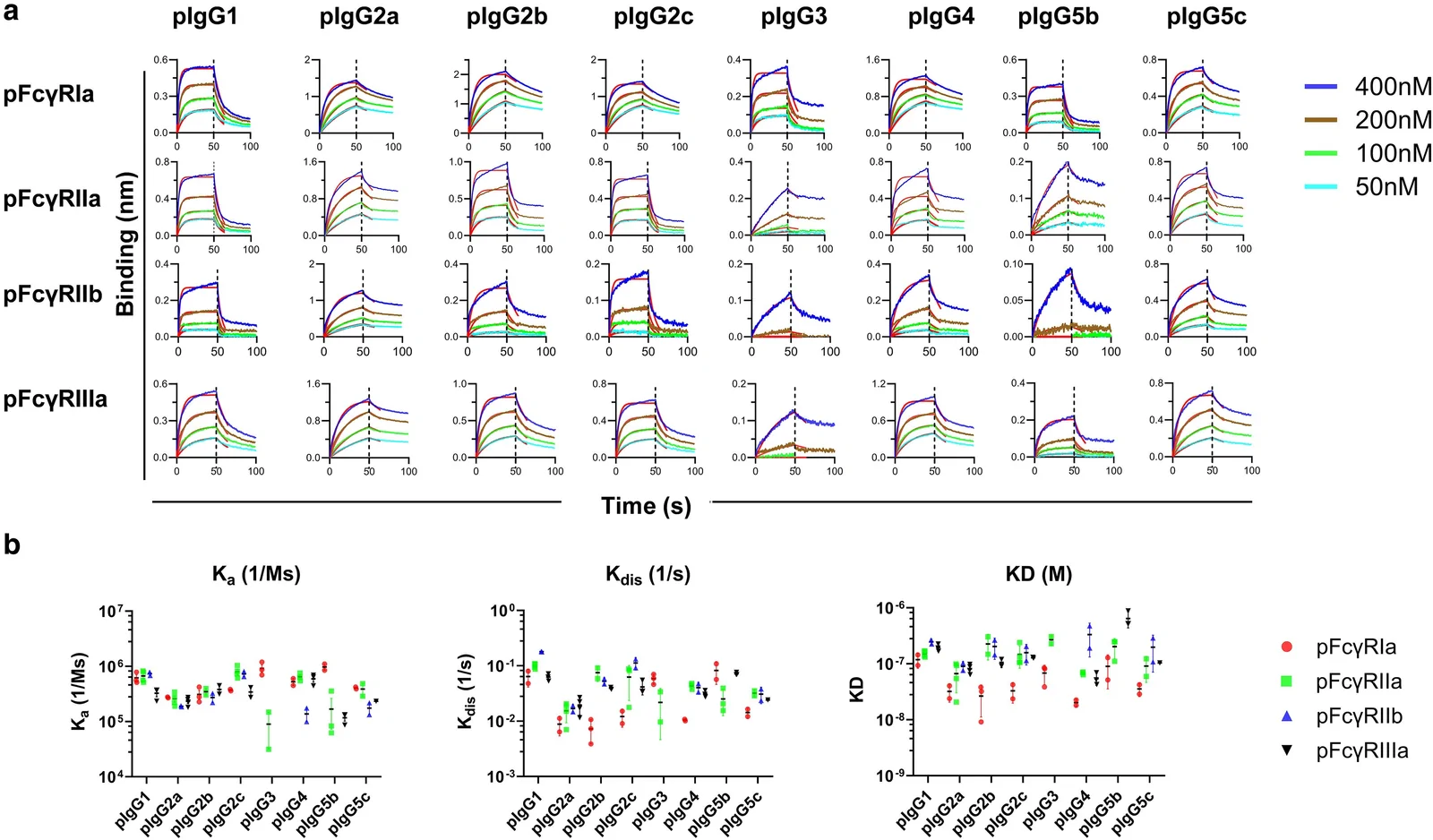
Binding of pIgG subclasses to pFcγRs. (a) BLI analysis of binding kinetics between pFcγRs and pIgG subclasses. Representative graphs show equilibration, association in pIgGs, and dissociation in buffer alone. Coloured lines show binding at 400, 200, 100, and 50 nM pIgG subclasses, and red lines show curve fitting by Octet analysis software. (b) The mean kinetics constants, K_a_, K_dis_, and KD between pFcγRs and pIgG subclasses. Each dot represents an independent experiment. The error bars represent the SEM, *n* = 2–4 independent experiments.

All pIgG subclasses bound pFcγRs in a concentration-dependent manner. Association kinetics for binding to the high-affinity receptor pFcγRIa were broadly comparable between subclasses, with association rate constant (K_a_) values ranging from 2.74 × 10^5^ to 9.75 × 10^5^ M^−1^ s^−1^ (Fig. 1b). Subclass-dependent differences were mainly driven by dissociation kinetics. Porcine IgG1, pIgG3, and pIgG5b dissociated rapidly and with a similar dissociation rate constant (K_dis_) (mean K_dis_ ≈ 6.84 × 10^−2^s^−1^), whereas pIgG2a, pIgG2b, pIgG2c, pIgG4, and pIgG5c exhibited ∼4-fold slower dissociation rates. This slower dissociation resulted in more stable binding and higher affinity interactions, with correspondingly lower affinity constants (KD) (mean KD ≈ 2.9 × 10^−8^ M) for pIgG2a, pIgG2b, pIgG2c, pIgG4, and pIgG5c (Fig. 1b and Table 1).

**Table 1:** Kinetic parameters of porcine FcγR-porcine IgG interactions.

		K_a_ (M^−1^s^−1^)	K_dis_ (s^−1^)	KD (M)
FcγRIa	pIgG1	6.08E+05	6.20E−02	1.19E−07
	pIgG2a	2.74E+05	8.51E−03	3.21E−08
	pIgG2b	2.99E+05	6.71E−03	2.65E−08
	pIgG2c	3.72E+05	1.17E−02	3.29E−08
	pIgG3	8.99E+05	5.77E−02	6.82E−08
	pIgG4	5.20E+05	1.05E−02	2.03E−08
	pIgG5b	9.67E+05	7.85E−02	8.98E−08
	pIgG5c	4.06E+05	1.41E−02	3.55E−08
FcγRIIa	pIgG1	6.57E+05	9.93E−02	1.52E−07
	pIgG2a	2.51E+05	1.42E−02	6.68E−08
	pIgG2b	3.46E+05	7.34E−02	2.26E−07
	pIgG2c	7.82E+05	5.03E−02	1.47E−07
	pIgG3	6.83E+04	1.81E−02	2.68E−07
	pIgG4	6.41E+05	4.35E−02	6.79E−08
	pIgG5b	1.23E+05	2.33E−02	2.02E−07
	pIgG5c	3.74E+05	3.19E−02	9.09E−08
FcγRIIb	pIgG1	7.34E+05	1.80E−01	2.46E−07
	pIgG2a	1.89E+05	1.68E−02	8.98E−08
	pIgG2b	2.68E+05	5.22E−02	2.04E−07
	pIgG2c	7.27E+05	1.10E−01	1.58E−07
	pIgG3	NB	NB	NB
	pIgG4	1.32E+05	3.98E−02	3.33E−07
	pIgG5b	NB	NB	NB
	pIgG5c	1.70E+05	3.00E−02	1.98E−07
FcγRIIIa	pIgG1	3.20E+05	6.21E−02	1.95E−07
	pIgG2a	2.31E+05	1.86E−02	8.17E−08
	pIgG2b	3.76E+05	3.93E−02	1.06E−07
	pIgG2c	3.37E+05	4.06E−02	1.29E−07
	pIgG3	NB	NB	NB
	pIgG4	5.84E+05	3.12E−02	5.45E−08
	pIgG5b	1.15E+05	7.19E−02	6.44E−07
	pIgG5c	2.33E+05	2.42E−02	1.04E−07

K_a_, association rate constant; K_dis_, dissociation rate constant; KD (M), affinity constant; NB, no binding.

In contrast to pFcγRIa, binding to pFcγRIIa was reduced for pIgG3 and pIgG5b (Fig. 1a). These subclasses also displayed low association rates, whereas pIgG1, pIgG2a, pIgG2b, pIgG2c, pIgG4, and pIgG5c exhibited broadly similar association kinetics (Fig. 1b). Porcine IgG1, pIgG2b, pIgG2c, and pIgG4 dissociated rapidly from pFcγRIIa (mean K_dis_ ≈ 7.02 × 10^−2^ s^−1^) compared with pIgG2a, pIgG3, pIgG5b, and pIgG5c, which dissociated more slowly (mean K_dis_ ≈ 2.36 × 10^−2^ s^−1^). Although pIgG4 showed relatively weak overall binding, its affinity was comparable to that of pIgG2a and pIgG5c, which displayed the strongest interactions with pFcγRIIa and the lowest affinity constants (mean KD ≈ 7.5 × 10^−8^ M).

The inhibitory receptor pFcγRIIb showed overall weaker interactions with pIgG subclasses. Binding to pIgG3 and pIgG5b was limited and insufficient to allow reliable calculation of kinetic parameters. Overall, the association kinetics of pIgGs with pFcγRIIb were like those observed for pFcγRIIa. Porcine IgG4 and pIgG5c displayed relatively slow association rates compared to the other subclasses. In contrast, pIgG1 and pIgG2c exhibited the fastest association rates (mean K_a_ ≈ 7.33 × 10^5^ M^−1^s^−1^), ∼4-fold higher than the remaining subclasses. However, pIgG1 and pIgG2c also dissociated more rapidly (mean K_dis_ ≈ 1.45 × 10^−1^s^−1^), again ∼4-fold faster than the other pIgGs.

The low-affinity receptor pFcγRIIIa also displayed limited interaction with pIgG3. Binding was detectable only at the highest concentration tested (400 nM), and kinetic parameters could not be reliably determined. Among all subclasses, pIgG5b showed the fastest association with pFcγRIIIa (K_a_ ≈ 1.17 × 10^5^ M^−1^s^−1^), resulting in the highest affinity interaction (KD ≈ 6.44 × 10^−7^ M). In contrast, pIgG2a and pIgG4 displayed the weakest binding affinities (KD ≈ 6.8 × 10^−6^ M), ∼10-fold lower than pIgG5b and 2-fold lower than the remaining subclasses.

Overall, these data demonstrate subclass-dependent differences in the interaction of pIgGs with pFcγRs. The pFcγRI displayed binding characteristics that differed from those expected of a classical high-affinity FcγR. pFcγRI consistently generated strong binding responses, particularly to IgG3, for which binding to the other porcine FcγRs was negligible. In addition, pFcγRI exhibited slightly higher affinities for IgG2a, IgG2b, IgG2c, IgG4, and IgG5c than the low-affinity receptors. The interactions with the low-affinity receptors pFcγRIIa, pFcγRIIb, and pFcγRIIIa were more variable and influenced by dissociation kinetics. Porcine IgG2 subclasses and pIgG5c generally exhibited slower dissociation and more stable interactions, while pIgG3 and pIgG5b showed weak or limited binding to several low-affinity receptors.

### Interactions and affinities of pIgG subclasses with pFcRn

The interaction of pIgG subclasses with pFcRn was also evaluated (Fig. 2a and b). Recombinant pFcRn was expressed as a soluble protein containing the extracellular domain and showed a characteristic double band, consistent with co-expression of FcRn with β2-microglobulin (β2M) as a heterodimeric complex fused to an Avi-tag and a C-terminal six-histidine tag (Supplementary Figure S1a and b).

**Figure 2:**
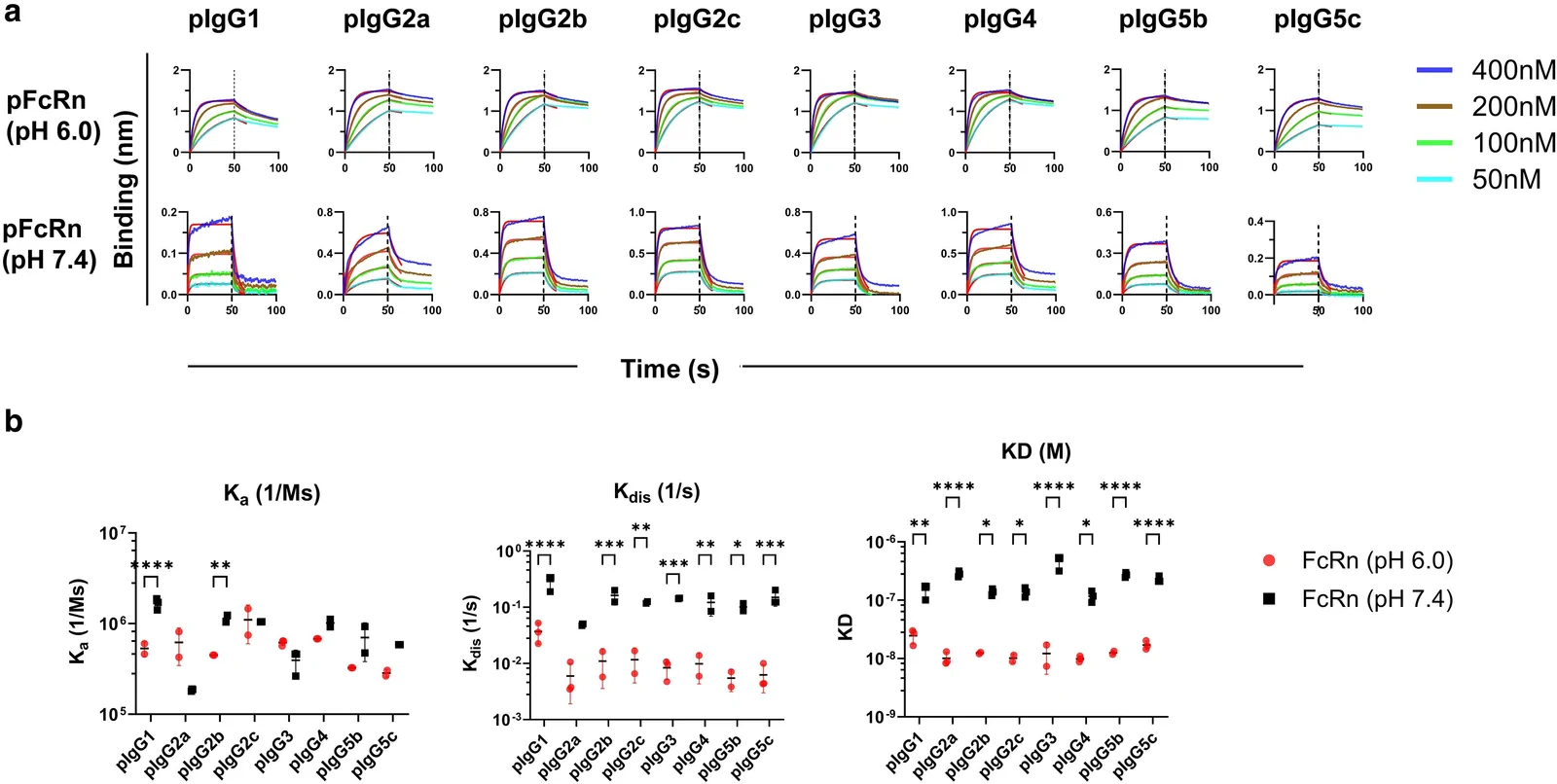
Binding of pIgG subclasses to pFcRn. (a) BLI analysis of binding kinetics between pFcRn at pH 6.0 and 7.4 and pIgG subclasses. Coloured lines show binding at 400, 200, 100, and 50 nM pIgG subclasses, and red lines show curve fitting by Octet analysis software. (b) The mean kinetics constants of pFcRn with pIgG subclasses. Each dot represents an independent experiment. The error bars represent the SEM, *n* = 2–4 independent experiments. Asterisks denote significance between indicated groups (*P < 0.05, **P < 0.01, ***P < 0.001, and ***P < 0.0001) and were analysed by two-way ANOVA and Šídák's multiple comparisons test.

Binding of pIgGs to pFcRn was pH dependent, with greater binding observed at acidic pH (6.0) compared with neutral pH (7.4). Although pIgG3 showed weak interactions with several low-affinity FcγRs, it had strong binding to pFcRn. At pH 6.0, all pIgG subclasses displayed similar association kinetics followed by relatively slow dissociation. In contrast, at pH 7.4, dissociation occurred rapidly for most subclasses, except for pIgG2a. All pIgG subclasses had broadly comparable association rate constants at pH 6.0 (mean K_a_ ≈ 4.98 × 10^5^ M^−1^s^−1^). At pH 7.4, pIgG1 showed the fastest association (K_a_ ≈ 1.65 × 10^5^ M^−1^s^−1^), whereas pIgG2a displayed a ∼9-fold lower association rate.

The most pronounced differences were observed in dissociation kinetics. The mean dissociation constant at pH 6.0 was 6.14 × 10^−3^s^−1^, compared with 1.33 × 10^−1^s^−1^ at pH 7.4, representing a ∼20-fold increase in dissociation at neutral pH. Among all subclasses, pIgG1 exhibited the fastest dissociation under both pH conditions. The slower dissociation at acidic pH resulted in much stronger binding affinities (mean KD ≈ 1.35 × 10^−8^ M) compared with neutral pH (mean KD ≈ 1.97 × 10^−7^M), corresponding to an overall 14-fold difference in affinity. The pH-dependent change in affinity was most pronounced for pIgG3 (35-fold) and least evident for pIgG1 (3-fold) (Fig. 2b and Table 2).

**Table 2:** Kinetic parameters of porcine FcRn-porcine IgG interactions.

			K_a_ (M^−1^ s^−1^)	K_dis_ (s^−1^)	KD (M)
pFcRn	pH 6	pIgG1	4.62E+05	1.26E−02	2.73E−08
		pIgG2a	4.25E+05	3.77E−03	8.88E−09
		pIgG2b	4.49E+05	5.75E−03	1.28E−08
		pIgG2c	7.46E+05	6.60E−03	8.85E−09
		pIgG3	6.04E+05	6.77E−03	1.12E−08
		pIgG4	6.79E+05	5.95E−03	8.77E−09
		pIgG5b	3.25E+05	3.84E−03	1.18E−08
		pIgG5c	2.84E+05	4.41E−03	1.54E−08
	pH 7.4	pIgG1	1.63E+06	2.54E−01	1.01E−07
		pIgG2a	1.83E+05	4.88E−02	2.66E−07
		pIgG2b	1.04E+06	1.25E−01	1.20E−07
		pIgG2c	1.05E+06	1.18E−01	1.13E−07
		pIgG3	3.48E+05	1.44E−01	4.13E−07
		pIgG4	9.22E+05	8.48E−02	9.20E−08
		pIgG5b	4.75E+05	1.17E−01	2.46E−07
		pIgG5c	5.87E+05	1.58E−01	2.11E−07

K_a_, association rate constant; K_dis_, dissociation rate constant; KD (M), affinity constant.

Together, these data demonstrate that interactions between pIgG subclasses and pFcRn are regulated by pH, consistent with the FcRn recycling mechanism. While all pIgG subclasses showed enhanced binding at acidic pH, clear subclass-dependent differences in dissociation kinetics and affinity were observed. Porcine IgG3 displayed enhanced pH-dependent binding to pFcRn despite weak interactions with several low-affinity FcγRs, suggesting that individual pIgG subclasses may differ not only in Fc-mediated effector functions but also in FcRn-dependent recycling and persistence.

### Interactions of hIgG subclasses with pFcγRs

We next investigated the interactions between hIgG and pFcγRs. hIgG subclasses containing kappa light chains were generated by isotype switching of the potent human 2-12C mAb, recognizing HA from H1N1pdm09 influenza A virus, into hIgG1, hIgG2, hIgG3, and hIgG4. Antibody purity and integrity of hIgGs were confirmed by SDS-PAGE analysis (Supplementary Figure S2a). To determine whether subclass switching affected antigen recognition, binding of the hIgGs to immobilized recombinant HA protein was assessed, confirming that each hIgG subclass retained antigen specificity and was selectively recognized by the corresponding anti-subclass antibody (Supplementary Figure S2b). The hIgGs were further characterized for their ability to activate the classical complement pathway using MDCK cells expressing H1 (MDCK-H1). Human IgG1 and hIgG3 mediated comparable levels of target cell lysis, while hIgG2 and hIgG4 did not activate the complement (Supplementary Figure S2c); in agreement with reported complement-activating properties of hIgG subclasses, hIgG1 and hIgG3 were potent activators, whereas hIgG2 and hIgG4 exhibited limited or no complement activation [23].

All hIgG subclasses showed measurable interactions with pFcγRs. Human IgG1 and hIgG3 showed the strongest binding to pFcγRIa, comparable to those for porcine pIgG2a, pIgG2b, pIgG2c, and pIgG4 (Fig. 3a and b and Table 3). However, direct comparisons between porcine and human IgGs should be interpreted cautiously because of intrinsic structural and functional differences between species. Association rate constants were broadly similar across the hIgG subclasses (mean K_a_ ≈ 2.32 × 10^5^ M^−1^s^−1^), whereas dissociation kinetics differed. Human IgG3 displayed the slowest dissociation rates to all pFcγRs (mean K_dis_ ≈ 4.45 × 10^−3^s^−1^), resulting in the strongest overall binding affinity. In contrast, hIgG2 dissociated more rapidly and consequently exhibited lower binding affinity. hIgG3 showed the highest affinity for pFcγRs (mean KD ≈ 4.40 × 10^−8^ M), whereas hIgG2 displayed the weakest interactions (mean KD ≈ 3.36 × 10^−7^ M) (Fig. 3b).

**Figure 3:**
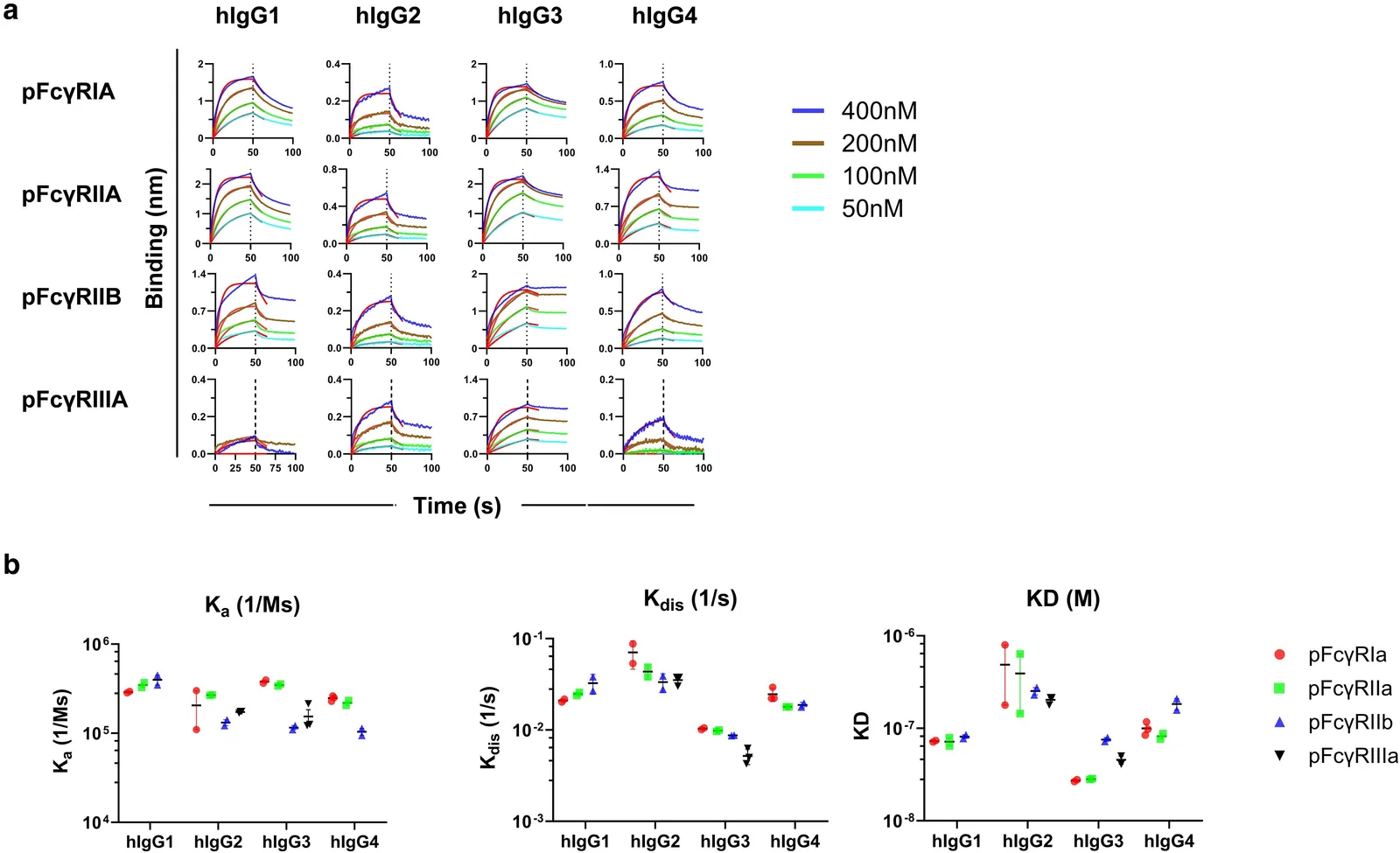
Binding of hIgG subclasses to pFcγRs. (a) BLI analysis of binding kinetics between pFcγRs and hIgG subclasses. Representative graphs show equilibration, association in hIgGs, and dissociation in buffer alone. Coloured lines show binding at 400, 200, 100, and 50 nM hIgG subclasses, and red lines show curve fitting by Octet analysis software. (b) The mean kinetics constants, K_a_, K_dis_, and KD between pFcγRs and hIgG subclasses. Each dot represents an independent experiment. The error bars represent the SEM, *n* = 2–4 independent experiments.

**Table 3:** Kinetic parameters of porcine FcγR-human IgG interactions.

		K_a_ (M^−1^ s^−1^)	K_dis_ (s^−1^)	KD (M)
FcγRIa	hIgG1	2.89E+05	2.10E−02	7.25E−08
	hIgG2	1.82E+05	6.84E−02	3.77E−07
	hIgG3	3.79E+05	1.03E−02	2.72E−08
	hIgG4	2.46E+05	2.44E−02	9.89E−08
FcγRIIa	hIgG1	3.47E+05	2.48E−02	7.14E−08
	hIgG2	2.65E+05	4.32E−02	3.04E−07
	hIgG3	3.48E+05	9.83E−03	2.83E−08
	hIgG4	2.19E+05	1.79E−02	8.19E−08
FcγRIIb	hIgG1	3.94E+05	3.20E−02	8.12E−08
	hIgG2	1.31E+05	3.29E−02	2.51E−07
	hIgG3	1.15E+05	8.71E−03	7.55E−08
	hIgG4	1.03E+05	1.87E−02	1.81E−07
FcγRIIIa	hIgG1	NB	NB	NB
	hIgG2	1.73E+05	3.50E−02	2.02E−07
	hIgG3	1.49E+05	5.12E−03	4.53E−08
	hIgG4	NB	NB	NB

K_a_, association rate constant; K_dis_, dissociation rate constant; KD (M), affinity constant; NB, no binding.

These data demonstrate that hIgG1, hIgG2, hIgG3, and hIgG4 subclasses can engage pFcγRs with distinct binding profiles. Similar to pIgGs, differences in binding affinity were driven primarily by dissociation kinetics rather than association rates.

### Interactions of hIgG subclasses with pFcRn

We also examined the binding of hIgGs to pFcRn (Fig. 4a and b and Table 4). Like the interactions of pIgGs, hIgG subclasses displayed rapid association kinetics and slow dissociation at pH 6.0. At pH 7.4, binding was markedly reduced for most subclasses, consistent with the pH-dependent FcRn recycling mechanism. However, hIgG3 exhibited a distinct kinetic profile characterized by both slower association and slower dissociation relative to the other subclasses. Quantitative analysis showed that the overall binding affinity at pH 6.0 (mean KD ≈ 1.3 × 10^−8^ M) was ∼10-fold higher than at pH 7.4 (mean KD ≈ 1.4 × 10^−7^ M). These findings show that hIgG subclasses interact efficiently with porcine FcRn in a pH-dependent manner like pIgGs, supporting the relevance of the pig model for evaluating the pharmacokinetics and persistence of human therapeutic mAbs.

**Figure 4:**
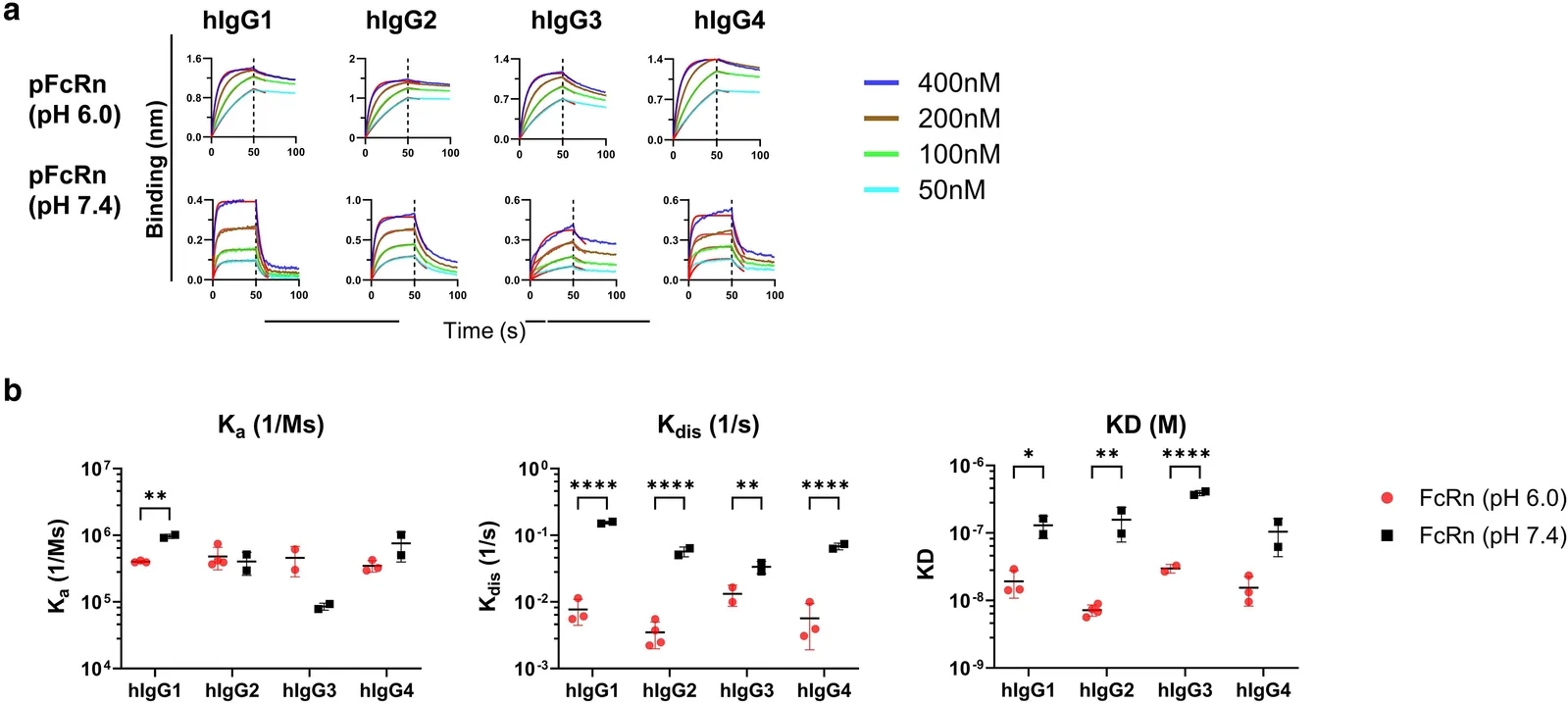
Binding of hIgG subclasses to pFcRn. (a) BLI analysis of binding kinetics between pFcRn at pH 6.0 and 7.4 and hIgG subclasses. Coloured lines show binding at 400, 200, 100, and 50 nM hIgG subclasses, and red lines show curve fitting by Octet analysis software. (b) The mean kinetics constants of pFcRn with hIgG subclasses. Each dot represents an independent experiment. The error bars represent the SEM, *n* = 2–4 independent experiments. Asterisks denote significance between indicated groups (*P < 0.05, **P < 0.01, ***P < 0.001, and ***P < 0.0001) and were analysed by two-way ANOVA and Šídák's multiple comparisons test.

**Table 4:** Kinetic parameters of porcine FcRn-human IgG interactions.

			K_a_ (M^−1^ s^−1^)	K_dis_ (s^−1^)	KD (M)
pFcRn	pH 6	hIgG1	4.0E+05	7.3E−03	1.8E−08
		hIgG2	4.6E+05	3.3E−03	7.1E−09
		hIgG3	4.3E+05	1.3E−02	3.0E−08
		hIgG4	3.4E+05	4.9E−03	1.4E−08
	pH 7.4	hIgG1	9.1E+05	1.5E−01	1.6E−07
		hIgG2	3.9E+05	5.7E−02	1.5E−07
		hIgG3	8.5E+04	3.3E−02	3.9E−07
		hIgG4	7.1E+05	6.8E−02	9.5E−08

K_a_, association rate constant; K_dis_, dissociation rate constant; KD (M), affinity constant.

### Fc-mediated function of hIgG in porcine leucocytes

As hIgG subclasses bound pFcγRs, we next investigated whether they could also bind porcine immune cell populations and trigger Fc-mediated effector functions. We assessed the binding of free hIgGs and virus-associated IgG immune complexes to porcine leucocytes. Monocytes are known to express FcγRIIa, FcγRIIb, and FcγRIIIa, whereas porcine NK cells predominantly express FcγRIIIa. Human IgG subclasses were incubated with H1N1pdm09 influenza A virus to generate immune complexes, and binding to porcine NK cells and monocytes was analysed by flow cytometry (Supplementary Figure S3a). Immune complexes with hIgGs bound porcine monocytes similarly to pIgGs. However, among the human subclasses, only hIgG2 showed detectable binding to NK cells (Fig. 5a; Supplementary Figure S4).

**Figure 5:**
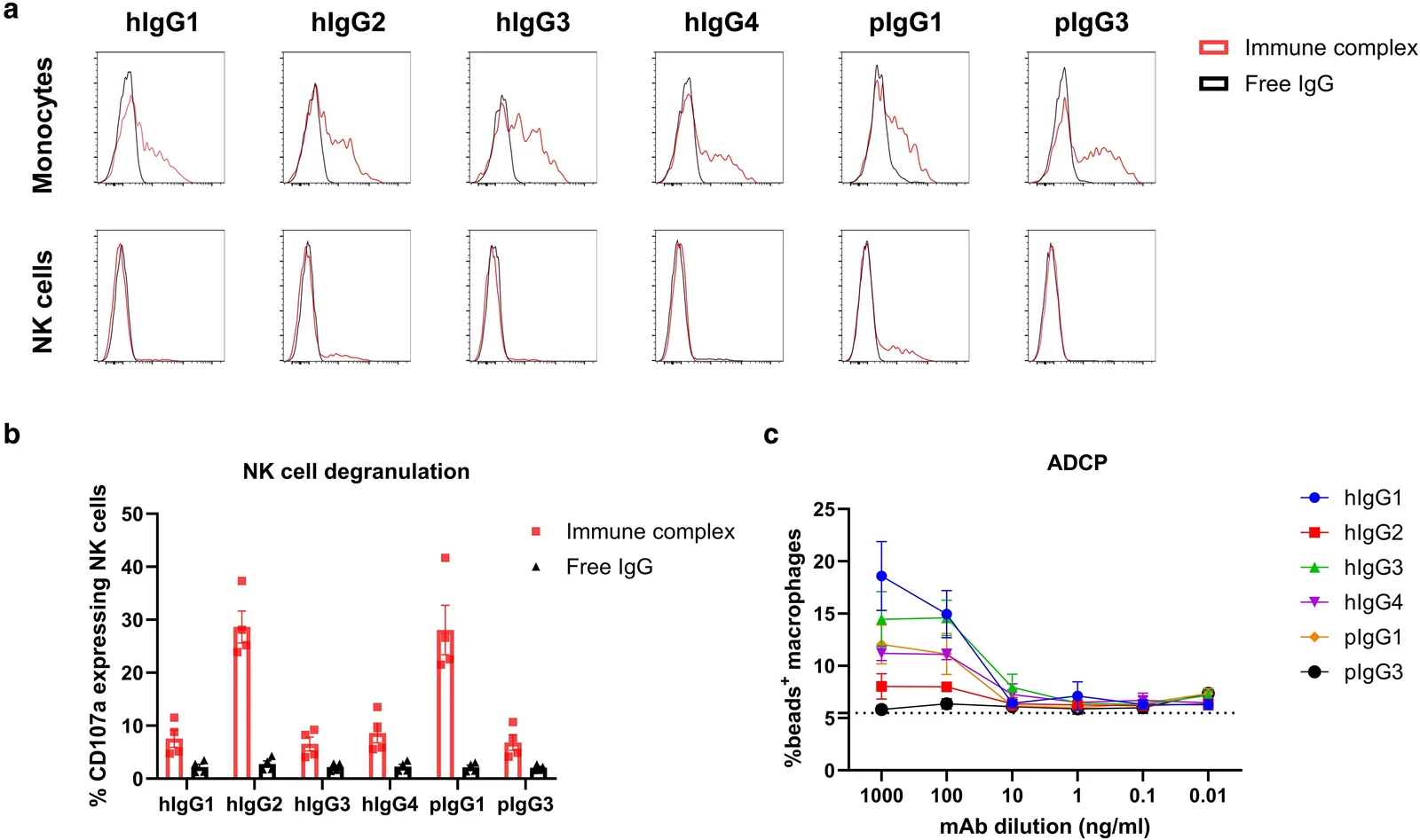
Binding of hIgG subclasses to porcine leucocytes and Fc-mediated functions. (a) Representative binding profile of free (red) and H1N1pdm09 virus immune complexed IgG subclasses (blue) to NK cells and monocytes. pIgG1 and pIgG3 were used as a control. Representative profiles from three experiments are shown (*n* = 3). (b) NK cell degranulation activity of hIgG subclasses. The error bars represent the SEM, *n* = 4. (c) Phagocytosis of fluorescence beads immune complexed with different human and porcine IgG subclasses by BAL macrophages. Dotted line shows the spontaneous phagocytosis of fluorescence beads by BAL macrophages. The error bars represent the SEM, *n* = 3.

NK cell degranulation, measured by surface expression of CD107a, was used as an indicator of NK cell cytolytic activity. Porcine peripheral blood mononuclear cells (PBMCs) were stimulated with porcine IL-2, IL-12, and IL-18 to generate activated NK cells. We previously demonstrated that all pIgG subclasses, except for pIgG3, mediate NK cell degranulation [19]. Therefore, we used pIgG1 and pIgG3 as positive and negative controls, respectively. Despite demonstrating strong binding to pFcγRIIIa in BLI, hIgG3 failed to induce NK cell degranulation (Fig. 3a and b). Surprisingly, only hIgG2 triggered NK cell degranulation, despite exhibiting relatively weaker binding affinity to pFcγRIIIa in BLI (Fig. 5b). However, BLI measured only monovalent interactions, whereas Fc-mediated effector functions were assessed in the context of immune complexes, where avidity effects are likely to contribute to receptor engagement and downstream effector functions. Furthermore, the degranulation induced by hIgG2 corresponded with its binding to NK cells (Fig. 5a). These results indicate that affinity measured by BLI does not necessarily translate into the intracellular signalling required for effector functions. Consistent with the absence of detectable interaction with pFcγRIIIa in both BLI and the NK cell binding assay, neither hIgG1 nor hIgG4 induced NK cell degranulation.

We next assessed the ability of hIgG subclasses to engage FcγRIIa on macrophages and mediate phagocytosis. Phagocytic activity was quantified as the proportion of viable macrophages internalizing FluoSphere beads (Fig. 5c). All hIgG subclasses mediated dose-dependent phagocytosis. Human IgG1 induced greater phagocytic activity than pIgG1, whereas hIgG2 exhibited weak activity. Consistent with previous observations, pIgG3 showed no detectable phagocytic activity [19]. Although both porcine and hIgGs bound monocytes efficiently, the hierarchy of phagocytic activity differed substantially, suggesting that FcγR binding alone does not fully predict downstream effector function.

In summary, these data demonstrate that hIgG subclasses can engage porcine immune cells and mediate Fc-dependent effector functions. However, pFcγR-binding affinity did not always correlate with functional activity, indicating that additional factors, beyond receptor engagement, such as receptor distribution, clustering, or intracellular signalling within the cell contribute to activation of downstream cellular responses.

### Fc domain sequence similarity and pFcγR-binding profiles of human and porcine IgG subclasses

We investigated whether similarities in pFcγR-binding profiles between hIgG and pIgG subclasses correlated with sequence similarity within the CH2–CH3 Fc domains. To address this, we compared a maximum likelihood (ML) phylogenetic tree based on Fc domain sequences with hierarchical clustering analysis of pFcγR-binding affinities (log10 KD) for hIgGs and pIgGs (Fig. 6).

**Figure 6:**
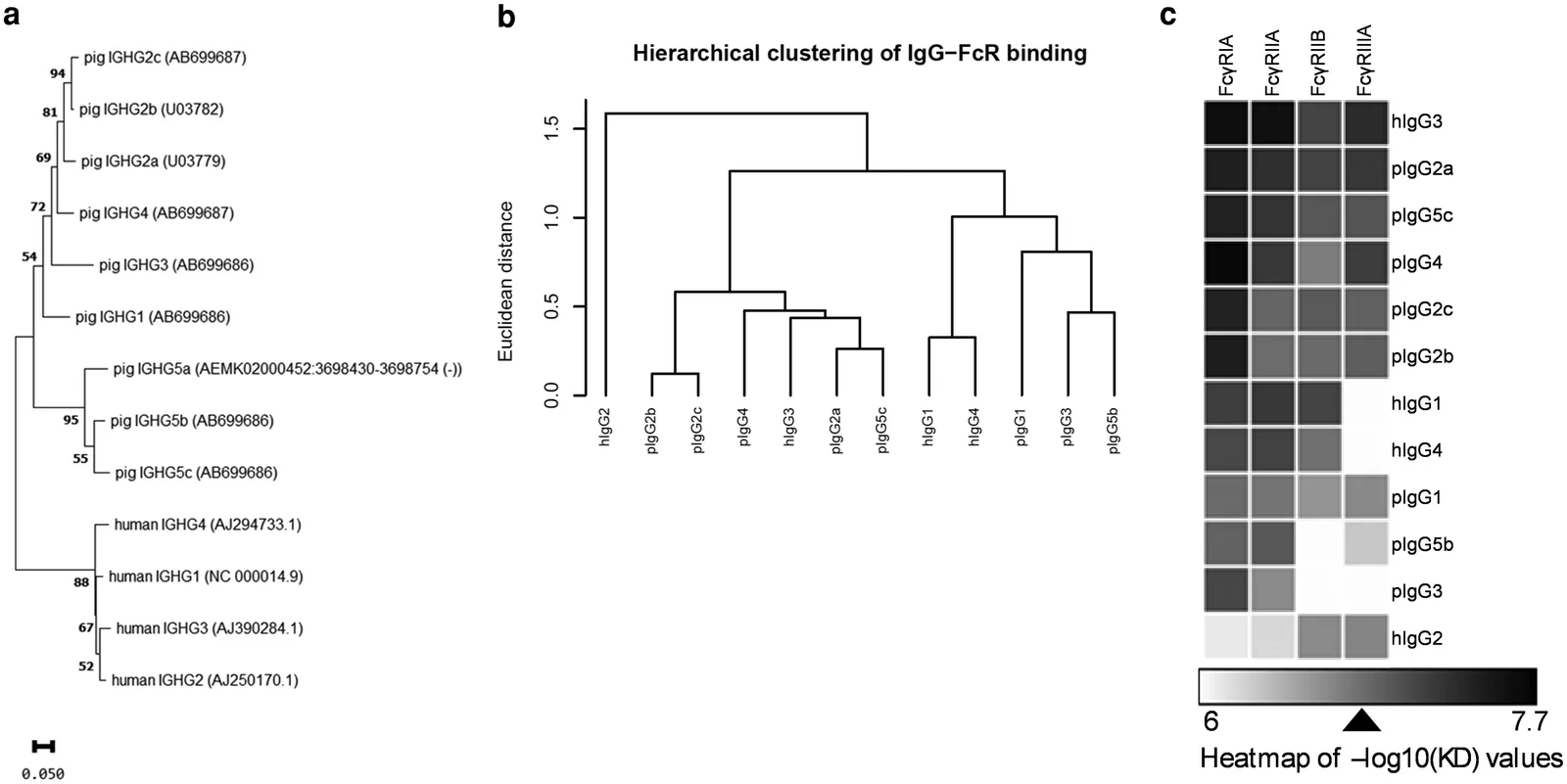
Comparative structural and functional divergence of human and porcine IgG. (a) ML phylogenetic tree of amino acid sequences spanning the CH2 and CH3 domains of human and pig IgG isotypes. ML phylogenetic tree was constructed in MEGA11 using default parameters and 1000 bootstrap iterations. Bootstrap support values ≥ 50% are shown. (b) Hierarchical clustering of human and pig IgG-FcγR binding profiles. Branch lengths represent Euclidean distance based on −log10(KD) values. The dendrogram was generated in R (v4.2.2) using Euclidean distance and average-linkage hierarchical clustering. (c) The heatmap representing the −log10 affinity constant (KD) values in different porcine and human IgG against porcine FcγRs.

The ML phylogenetic tree showed clear species-specific clustering of human and porcine IgG subclasses. Within the pIgGs, *pIGHG2B* and *pIGHG2C* clustered closely together, as did *pIGHG5B* and *pIGHG5C*, indicating strong sequence similarity within these subclasses (Fig. 6a). In contrast, the pFcγR-binding dendrogram grouped IgGs according to functional receptor-binding similarity, which in several cases differed from their phylogenetic relationships. For example, pIgG2b and pIgG2c clustered together in both analyses, suggesting that similar CH2–CH3 sequences are associated with similar FcγR-binding properties. However, this relationship was not maintained for pIgG5b and pIgG5c, which clustered separately in the functional analysis despite close phylogenetic relatedness.

Interestingly, the FcγR-binding dendrogram also indicated that certain human subclasses, including hIgG1 and hIgG4, clustered functionally with specific porcine subclasses such as pIgG1, pIgG3, and pIgG5b. The most striking discrepancy was observed for hIgG2. Although hIgG2 clustered with the other human subclasses in the phylogenetic tree, it formed a distinct and highly separated branch in the FcγR-binding dendrogram, indicating a unique functional binding profile (Fig. 6b and c). Similarly, although *pIGHG5a*, *pIGHG5b*, and *pIGHG5c* clustered together phylogenetically, functional clustering separated pIgG5b from pIgG5a and pIgG5c, with pIgG5b instead grouping with pIgG3.

Together, these analyses demonstrate that FcγR-binding phenotypes are only partially predicted by Fc domain sequence similarity. Closely related IgG subclasses can display very different receptor-binding profiles, indicating that relatively small sequence differences within the Fc region may substantially influence FcγR interactions and downstream effector functions.

## Discussion

In this study, we characterized the interactions of porcine and human IgG subclasses targeting the same epitope on influenza HA with pFcγRs and FcRn and evaluated the ability of human IgGs to mediate Fc-dependent effector functions in porcine cells. Porcine IgG subclasses showed distinct binding profiles across pFcγRs, with the greatest variability observed for the low-affinity receptors pFcγRIIa, pFcγRIIb, and pFcγRIIIa. Furthermore, the distinction between high- and low-affinity FcγRs in pigs may be less pronounced than that described for human FcγRs. Notably, human FcγRI does not bind all IgG subclasses equally, with human IgG2 showing no interaction despite FcγRI being classified as a high-affinity receptor [24]. Differences in receptor interaction were driven predominantly by dissociation kinetics rather than association rates, indicating that the stability of FcγR engagement is likely a major determinant of Fc-mediated activity in pigs. Similar observations have been reported in humans and mice, where slower dissociation kinetics prolong FcγR engagement, which can promote receptor clustering and enhance downstream signalling and effector functions [25]. The recent publication by Van der Weken *et al*. has demonstrated that porcine IgG effector activity is linked to discrete Fc sequence motifs. The revised pIgG4 family, IgG2c in this case, and selected IgG4 variants, contains a conserved G234-P235 lower-hinge motif associated with enhanced FcγR engagement and innate immune-cell activation [26]. The relatively stable interactions of pIgG2 with several pFcγRs suggest that these subclasses may have greater capacity to induce Fc-mediated effector functions *in vivo*. In contrast, the P329L and A330S substitutions present in pIgG3 are predicted to disrupt the FcγR and C1q-binding interface [26], consistent with the weak or limited interaction with several low-affinity receptors, and limited functional activity observed for this subclass [19]. Together, these findings demonstrate that discrete Fc sequence differences, alongside receptor-binding kinetics, are important determinants of porcine IgG function.

The interactions between pIgGs and pFcRn demonstrated the characteristic pH dependence for FcRn-mediated recycling. All subclasses bound more strongly at acidic pH than at physiological pH, consistent with the established FcRn pathway that protects IgG from lysosomal degradation and extends serum half-life [27]. Interestingly, pIgG3 displayed relatively weak interactions with low-affinity pFcγRs while maintaining robust strong pH-dependent binding to pFcRn, suggesting that Fc-mediated effector function and FcRn-mediated recycling may be independently regulated in pigs. This observation is supported by sequence analyses showing that, despite mutations affecting FcγR engagement, including alterations with the hinge and FcγR-interacting regions, the residues and motifs predicted to mediate FcRn binding mainly in CH2-CH3 interface region remain largely conserved in pIgG3 and are comparable to those found in other porcine IgG subclasses [26].

A major objective of this study was to determine whether hIgG1, hIgG2, hIgG3, and hIgG4 subclasses interact productively with the pFcγRs system. Overall, the kinetic parameters of hIgGs were relatively similar across the different pFcγRs, in contrast to interactions reported for human FcγRs, where hIgGs bind the high-affinity receptor FcγRI substantially more strongly than the lower-affinity receptors [24]. Nevertheless, subclass-dependent differences were observed in the porcine system. Human IgG3 showed the strongest and most stable interactions with pFcγRs, whereas hIgG2 displayed comparatively weaker binding.

Importantly, hIgG1 and hIgG4 did not bind pFcγRIIIa, consistent with the findings of Egli *et al.* who reported that hIgG1 failed to interact with pFcγRIIIa, whereas pIgG1a bound efficiently [12]. Similar observations were reported by Morgan *et al.* demonstrating that hIgG1 does not effectively engage pFcγRIIIa [28]. The molecular basis for this species-specific difference appears to lie within the receptor-binding interface. Engagement of FcγRIII depends on residues within the lower-hinge region of IgG, including the conserved ‘LLGG’ motif that is critical for receptor interaction. Although this motif is present in hIgG1, pFcγRIIIa differs structurally from its human counterpart within the FG loop, containing an ‘IIK’ motif rather than the hydrophobic residues found in human FcγRs. Structural modelling suggests that the presence of a positively charged lysine residue disrupts stabilizing interactions with the human IgG Fc region, thereby impairing receptor engagement [28]. In contrast, Bhatti *et al*. reported binding of hIgG1 to pFcγRIIIa. However, this interaction was only detected at a high antibody concentration (40 μM), much greater than the concentration used in the present study (400 nM) [29]. Furthermore, receptor binding was enhanced following introduction of the Fc-engineering mutations S239D/I332E (SD/IE), which are known to increase FcγRIIIa affinity (Bhatti *et al*., 2019). Together, these findings suggest that wild-type hIgG1 interacts only weakly with pFcγRIIIa and that detectable binding may require either high antibody concentrations or Fc modifications that enhance receptor engagement.

Functional studies further supported these binding data. Human IgG1 did not induce NK cell degranulation, consistent with its lack of detectable interaction with pFcγRIIIa. Surprisingly, only hIgG2 induced NK cell degranulation despite exhibiting relatively weak affinity for pFcγRIIIa. Conversely, hIgG3 displayed strong binding to pFcγRIIIa in BLI but failed to activate NK cells. This discrepancy may reflect differences in receptor presentation between the two systems, as recombinant pFcγRIIIa immobilized in BLI may expose binding interfaces that are less accessible when the receptor is expressed in its native membrane on NK cells. Furthermore, differences in glycosylation between recombinant and native receptors may influence receptor recognition and contribute to the lack of correlation between biophysical binding and cellular activity, consistent with previous studies demonstrating a role for FcγRIIIa glycosylation in modulating IgG interactions [30]. However, such effects were not observed with pIgGs suggesting that this discordance is specific to the interaction between hIgG3 and porcine receptor. These findings contrast with observations in humans, where hIgG1 and hIgG3 are typically the most effective subclasses at engaging FcγRIIIa and mediating ADCC [31]. Although pigs and humans possess homologous FcγR systems, the CH2 and CH3 Fc domains of human and pig IgG exhibit limited sequence homology and strong species-specific homogenization driven by concerted evolution, as shown by phylogenetic analyses in pigs, goats, cattle, sheep, humans, and mice [28, 32]. Thus, while FcγRs are genomically conserved across species, pFcγR-IgG interactions appear structurally and functionally distinct from the human system, particularly for FcγRIIIa-mediated effector functions such as ADCC. The difference between *in vitro* interaction and functional assays indicates a limitation of relying solely on *in vitro* binding techniques such as surface plasmon resonance and BLI. Although these methods provide high-resolution kinetic measurements, they do not capture the complexity of receptor clustering, membrane organization, receptor co-expression, ligand valency, cellular context, and downstream signalling through ITAM pathways required for functional activation [33, 34]. Such differences may limit the ability of pigs to accurately predict FcγRIIIa-dependent functions of human therapeutic antibodies, particularly NK cell-mediated ADCC. The development of humanized minipigs expressing human IgG repertoires and the Fc receptor system may provide more appropriate models to analyse Fc-mediated functions of human mAbs [35, 36].

In contrast to the NK cell responses, ADCP was mediated by all human IgG subclasses. All hIgGs interacted with pFcγRIIa, a receptor primarily associated with phagocytic activity, and induced phagocytosis in porcine macrophages. The hierarchy of activity broadly mirrored that observed in humans, with hIgG1 and hIgG3 generally more effective than hIgG2 and hIgG4 at engaging FcγRs and mediating effector functions [37–39]. The preservation of this functional hierarchy suggests that aspects of FcγR-mediated phagocytic biology are conserved between pigs and humans, despite the species-specific differences observed for FcγRIIIa-dependent responses.

Human IgGs also interacted efficiently with pFcRn in a pH-dependent manner, comparable to pIgGs, in agreement with other studies [7, 40]. We previously demonstrated that the serum half-life of the 2-12C hIgG1 antibody in pigs was comparable to that of the porcine pb27 IgG1 antibody [9], and the current data provide a mechanistic explanation for this observation. Efficient interaction of hIgGs with pFcRn is likely to contribute to the comparable pharmacokinetics observed *in vivo* and further supports the utility of pigs as a relevant large animal model for antibody delivery and pharmacokinetic studies. Species-specific differences in FcRn interactions are well recognized. For example, hIgG1 interacts with cynomolgus monkey FcRn with ∼2-fold higher affinity than with hFcRn and binds mouse and rat FcRn with ∼10-fold higher affinity [18]. The ability of pFcRn to bind hIgGs in a physiologically relevant manner therefore represents an important advantage of the pig model.

This study has several limitations. Binding analyses were performed using soluble recombinant Fc receptors and human and porcine IgGs which may not fully recapitulate the complexity of receptor organization, avidity effects, and immune complex formation on primary cells *in vivo*. In addition, Fc glycosylation patterns, which are known to influence FcγR binding and Fc-mediated function, were not specifically analysed. Future studies investigating the structural and glycosylation-dependent determinants of FcγR recognition, combined with comparative computational analyses of human and porcine IgGs, could provide valuable mechanistic insight into how sequence variation shapes receptor-binding interactions.

In summary, this study provides a comprehensive characterization of porcine and human IgG interactions with pFcγRs and pFcRn, demonstrating that human IgGs can engage the porcine Fc receptor system and mediate Fc-dependent effector functions. We identified IgG subclass-specific differences in receptor binding and function and showed that Fc domain sequence similarity alone is not sufficient to predict FcγR engagement or biological activity. While key aspects of Fc biology are conserved between humans and pigs, important differences in FcγR-mediated responses limit direct translational extrapolation. Our findings support the pig as a relevant model for evaluating the pharmacokinetic properties of human monoclonal antibodies. However, we also demonstrate the need for caution when assessing therapeutic antibody efficacy that depends on Fc-mediated effector functions, particularly FcγRIIIa-dependent mechanisms such as ADCC. These results indicate the importance of integrating biophysical, structural, and functional analyses when evaluating antibody activity between species and will contribute to improving the predictive value of preclinical animal models in informing the rational design of next-generation antibody therapeutics.

## Methods

### Generation of porcine and human IgG subclasses

Porcine (pb27) and human (2-12C) anti-influenza monoclonal antibodies targeting the K130 epitope of HA from the H1N1pdm09 virus were used to generate panels of porcine and human IgG subclasses [21, 41].

Recombinant porcine pb27 IgG subclasses, IgG1, IgG2a, IgG2b, IgG2c, IgG3, IgG4, IgG5b, and IgG5c, were generated as described previously [19]. Briefly, the VDJ variable region of pb27 heavy chain was isotype-switched into eight IgG subclasses using pNeoSec vectors carrying corresponding porcine IGH constant regions and co-transfected with IgL vector carrying pb27 VJ light chain for expression.

The human 2-12C IgG1 antibody was isotype-switched to human IgG1, IgG2, IgG3, and IgG4 subclasses as described for generation of porcine IgG subclasses [19]. Human IGH and IGκ expression vectors were made using pNeoSec cassette backbone as previously described [21]. Briefly, gene sequences for the human *IGHG1* (UniProt ID: P01857-1), *IGHG2* (UniProt ID: P01859-1), *IGHG3* (UniProt ID: P01860-1), *IGHG4* (UniProt ID: P01861-1), and IGκ (UniProt ID: P01834) constant regions were obtained from public databases and ordered as synthetic gene fragments (Twist Bioscience) to clone into pNeoSec expression vectors. The gene fragments encoding 2-12C VDJ and VJ variable regions (Twist Bioscience) were then correspondingly cloned into pNeoSec-Human-IGHG1, pNeoSec-Human-IGHG2, pNeoSec-Human-IGHG3, pNeoSec-Human-IGHG4, and pNeoSec-Human-IGκ vectors, under the μ-phosphatase secretion leader and in frame to a downstream constant region for expression of recombinant human antibodies. The heavy and light chain pairs for each IgG subtype were then transiently co-expressed in Expi293F™ cells followed by purification on an ÄKTA pure™ 25 using 5 ml Protein G chromatography (Cytiva, Amersham, UK) [42].

### Generation of recombinant soluble porcine FcγRs

Porcine FcγRs and pFcRn were generated as soluble proteins using the method for bovine FcγRs as described previously [20]. Nucleotide coding sequences for porcine *FCGR1A* (Chr 4: 99669910–99660937), *FCGR2A* (Chr 4: 89590861–89602081), *FCGR2B* (Chr 4: 89486340–89505013), *FCGR3A* (Chr 4: 89560677–89568779), *FCGRT* (FcRn; Chr 6: 54596273–54605669), and *β2-microglobulin B2M* (Chr 1: 126963373–1269676998) were manually annotated within the Babraham pig genome assembly, TPI_Babraham_pig_v1 [43], using Artemis v17.0.1 [44] and informed using Iso-Seq data (BioProject accession: PRJNA351265) [45]. The gene sequences for the extracellular domains of pFcγRIA, pFcγRIIA, pFcγRIIB, pFcγRIIIA, and pFcRn fused to Avi tag and a six Histidine (6xHis) tag at the C-terminus were codon-optimized for expression in Expi293F™ cells and ordered as synthetic DNA fragments (Integrated DNA Technologies). The constructs were cloned into pNeoSec-derived expression vector under the heterologous secretion leader (μ-phosphatase) leader sequence through Infusion cloning using ClonExpress II One Step Cloning Kit (Vazyme) as per manufacturer instructions. The pFcγRs were then transiently transfected into Expi293F™ cells (Gibco™ A14527) using Polyethylenimine (40 kD) MAX® (Polysciences). Porcine FcRn and B2M constructs were co-transfected to express the heterodimer pFcRn protein. The cell culture supernatants carrying soluble proteins were harvested after 3 days of incubation before processing for Immobilized Metal Affinity Chromatography (IMAC) purification. The supernatants were buffer exchanged to phosphate-buffered saline (PBS) using tangential flow filtration to remove media components that interfere with his-tag purification. In brief, one Sartorius vivaflow 200 cm^2^, 10 kDa molecular weight cut-off (MWCO) hydrosart membrane (Sartorius Cat: VF20H0) was conditioned in PBS and then the supernatant was concentrated to 100 ml retentate volume and diafiltered using 500 ml of PBS (5 diavolumes for >99% solute removal) following the manufacturer's instructions. The buffer exchanged supernatant was purified in bind-elute mode by 5 ml HisTrap FF column chromatography (Cytiva Cat: 17528601) using an ÄKTA pure™ 25. The buffer exchanged supernatant was adjusted to 500 mM sodium chloride and 20 mM imidazole and loaded onto the equilibrated column at 5 ml/min. Following a 40 mM imidazole wash step, the purified protein was eluted in 200 mM imidazole buffer. The purified protein was buffer exchanged to PBS using a 10 kDa MWCO dialysis cassette (Thermo Cat: 66830) following the manufacturer's instructions. If required, the protein was concentrated using an Amicon 10 kDa MWCO centrifugal filter (Millipore UFC 501024) following the manufacturer's instructions. Confirmation of purity was determined by SDSPAGE (4–12% Bis-Tris/MES Thermo Cat: NP0323BOX) with Coomassie staining (InstantBlue, Abcam Cat: ab119211).

### BLI

BLI was performed on an Octet R8 instrument (Sartorius) to characterize mAb–FcγR interaction kinetics. All steps were conducted at 30°C with microplate agitation at 1000 rpm and a data acquisition rate of 10.0 Hz. Kinetics buffer (Sartorius) was used to dilute reagents, hydrate streptavidin biosensors, and establish baselines. Recombinant pFcγRs were biotinylated and immobilized onto streptavidin biosensors (Sartorius Cat: 18-5019) at 100 nM to a binding response of 1.0 nm, followed by a 60 s buffer dip and a 10 s baseline step. mAbs were associated at 400, 200, 100, 50, and 0 nM for 50 s and then dissociated for 100 s. For FcRn, receptor loading and baseline were performed at pH 7.4; dissociation was carried out at either pH 6.0 or pH 7.4 in phosphate-based assay buffer (100 mM sodium phosphate, 150 mM NaCl, 0.05% Tween-20). Data were baseline-aligned, interstep-corrected to dissociation, and Savitzky–Golay smoothed. Binding sensograms were analysed using a 1:1 interaction model in Octet Analysis Studio v13.0.3.52 (Sartorius). The dissociation phase exhibited biphasic behaviour suggesting the presence of complex binding behaviour that may arise from heterogeneous binding states, receptor avidity, or multiple interaction conformations, that was not adequately described by a single 1:1 model over the full dissociation interval. Therefore, kinetic fitting was performed using the initial 15 s of the dissociation phase, capturing the primary dissociation component while excluding the slower secondary phase. The resulting kinetic parameters represent values derived from the initial dissociation component and were used for comparative assessment of FcγR binding between samples. To ensure data quality, the kinetic parameters were acceptable when the errors for KD, K_a_, and K_dis_ were below 10% of KD, K_a_ and K_dis_, with *R*^2^ > 0.98 throughout.

### Enzyme-linked immunosorbent assay

Isotype enzyme-linked immunosorbent assay was used to confirm human IgGs. Maxisorp 96-well plates (Thermo Scientific, UK) were coated with 100 µl of 1 µg/ml recombinant influenza A virus HA1 protein (A/Eng/195/2009) in carbonate-bicarbonate (CBC) buffer overnight at 4°C. The plates were washed three times with PBS containing 0.05% Tween-20 (PBS-T) and blocked with 4% (w/v) skimmed milk in PBS-T for 1 hour at room temperature. Human IgG1, hIgG2, hIgG3, and hIgG4 mAbs were diluted to 1 µg/ml in blocking buffer, added at 100 µl per well, and incubated for 1 hour at room temperature with shaking. After washing three times, mouse anti-human IgG1 (Invitrogen, Cat: 10592489), IgG2 (Invitrogen, Cat: 0727043), IgG3- horseradish peroxidase (HRP) (Invitrogen, Cat: 10660094), and IgG4-HRP (Invitrogen, Cat: 10192478) secondary antibodies were diluted either 1:1000 or 1:4000 in blocking buffer, added at 100 µl per well, and incubated for 1 hour at room temperature. To detect hIgG1 and hIgG2, goat anti-mouse HRP (DAKO Cat: P0447) was added at a 1:3500 dilution in blocking buffer after a further three washes. Plates were washed three times before being developed with 50 µl of 3,3′,5,5′-tetramethylbenzidine (TMB) substrate (BioLegend) per well. The reaction was stopped with 50 µl of 1 M H_2_SO_4_, and absorbance was read at 450 and 630 nm using a Biotek ELx808 reader.

### Complement-dependent cytotoxicity

The ability of hIgGs and pIgGs to activate the complement pathway was assayed using pre-adsorbed rabbit low-tox-H complement (Cedarlane Laboratories Cat: CL3305-R) as described previously [19]. Briefly, serially diluted IgGs were incubated with MDCK-SIAT1 cells stably expressing HA1 from influenza A virus H1N1 (A/Eng/195/2009) (MDCK-H1) and with rabbit complement sera at a final concentration of 5% for 2 hours at 37°C. Plates were centrifuged at 420 × g for 4 minutes, after which 50 µl of supernatant was transferred to a flat-bottom plate. LDH substrate (50 µl) was added, and absorbance was measured at 490–630 nm at 1-minute intervals using a BioTek ELx808 reader. Complement activation was quantified as the percentage lysis of target cells relative to the maximum lysis induced by 1% Triton-X100.

### NK cell degranulation

Degranulation of NK cells was assessed using a modified flow cytometry based assay using surface mobilization of CD107a as a readout as described previously [19]. PBMCs were stimulated overnight with recombinant porcine IL-2 (20 ng/ml; Kingfisher Biotech), IL-12 (25 ng/ml; R&D Systems), and IL-18 (100 ng/ml; R&D Systems). Maxisorp 96-well plates were coated with 5 µg/well of recombinant HA1 protein (A/Eng/195/2009) in CBC buffer overnight at 4°C, then washed and blocked with 1% BSA in PBS for 1 hour at room temperature. Human IgGs at 10 µg/ml was added to HA-coated and uncoated wells and incubated for 1 hour at room temperature. Stimulated PBMCs were washed with and added into the HA coated and uncoated well together with anti-CD107a-FITC (clone 4E9/11; IgG1, Bio-Rad; 4 µg/ml), brefeldin A (GolgiPlug, BD Biosciences), and monensin (GolgiStop, BD Biosciences) and incubated for 5 hours at 37°C. Cells were transferred to a U-bottom plate, stained with a live/dead near-infrared viability dye (Thermo Fisher), anti-CD3ε (clone BB23-8E6-8C8, BD Biosciences), and anti-CD8α (clone 76-2-11, BD Biosciences) for 15 minutes at 4°C, and were then fixed with 4% paraformaldehyde. Data were acquired on a MACSQuant 10 flow cytometer (Miltenyi Biotec) and analysed using FlowJo v10 (BD Life Sciences). NK cells were defined as CD3^−^CD8α^+^ and the percentage expressing CD107a was recorded.

### ADCP

Phagocytic ability of hIgGs were assessed using Red FluoSpheres Neutravidin-Labeled Microspheres (Thermo Fisher Scientific Cat: F8775) as described previously [46]. Briefly, FluoSpheres beads were coated with biotinylated HA (25 µl at 500 µg/ml) for 2 hours at 37°C, washed twice with PBS and diluted in 500 µl of PBS. In a 96-well plate, 10 µl of beads conjugated with HA (beads-HA complex) was incubated with 20 µl of hIgGs at different concentrations for 2 hours at 37°C to form immune complexes (Beads-HA + hIgGs). Fifteen microliters of Beads-HA + hIgGs complexes was then added to 75 µl of bronchoalveolar lavage cells (500 000 cells/well) isolated from healthy naive pigs as effector cells and incubated at 37°C for 1.5 hours. Cells were washed, stained with a live/dead near-infrared viability dye (Thermo Fisher Scientific), CD163-FITC (Bio-Rad: Clone 2A10/11) for 15 minutes at 4°C in the dark, and fixed with 4% PFA. Data were acquired on a MACSQuant 16 flow cytometer (Miltenyi Biotec) and analysed using FlowJo v10 (BD Life Sciences). ADCP was quantified as the percentage of live macrophages double positive for Red FluoSpheres beads.

### Leucocyte binding assay

To study the interaction of the different human and porcine IgG subclasses with porcine leucocytes that natively express FcγRs, immune complexes were generated as previously described [19]. Briefly, 10 µg/ml of hIgGs and pIgGs was incubated with H1N1pdm09 (A/Eng/1353/2009) for 30 minutes at 37°C to generate immune complexes. The resulting complexes were then incubated with PBMCs for 1 hour at 37°C. The cells were washed and stained with Live/Dead Near Infra-Red Stain, and immune complex binding was detected by staining with rabbit-anti pig IgG (H + L)-FITC (Thermo Fisher Scientific) for pIgG immune complexes and mouse anti-human IgG Alexa flour 488 (BioLegend, Clone: HP6017) for human immune complexes. Binding to NK cells and monocytes was determined by co-staining with CD3^−^CD8a^+^ for NK cell and CD172a^+^CD14^+^ for monocytes and acquired on a MACSQuant 16 flow cytometer (Miltenyi Biotec). The percentage of NK cells or monocytes binding IgG was analysed using FlowJo v10 (BD Life Sciences).

### Statistical analysis

GraphPad Prism 11.0.2 (GraphPad Software, San Diego, CA, USA) was used for all statistical analyses. The data set was analysed by two-way ANOVA and Šídák's multiple comparisons test. Significant differences are presented on each graph (*P  *<* 0.05, **P  *<* 0.01, ***P  *<* 0.001, ****P  *<* 0.0001).

## Supplementary Material

kyag018_Supplementary_Data

## Data Availability

The original contributions presented in the study are included in the article/Supplementary material. Further inquiries can be directed to the corresponding author.

## Permission to reproduce

Graphical abstract was created in BioRender. Paudyal, B. (2026) https://BioRender.com/0ngs62h.
