# Supplementary material for: Human and porcine IgG subclasses differentially engage porcine Fc receptors
Source: Discov Immunol. 2026 Sep 21;5(1):kyag018. doi: 10.1093/discim/kyag018 (PMC13643491; doi:10.1093/discim/kyag018)
Supplement: Supplementary file 1 [file kyag018_supplementary_data.pdf]

Supplementary Figure S1

A

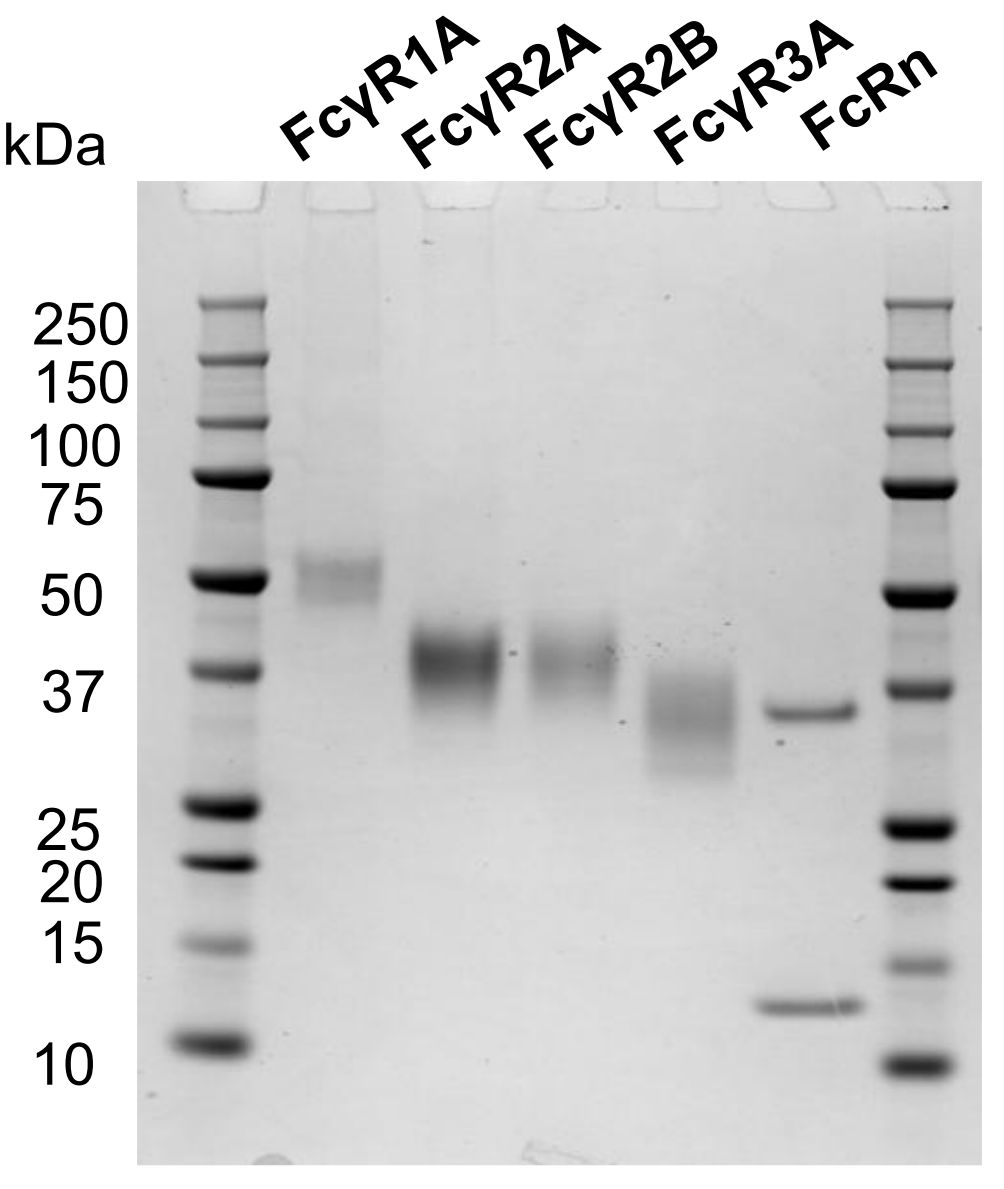

B

Protein	Predicted weight (kDa)	Protein Yield (mg)	Expression scale (mL)
FcγR1A	33.38	1.23	300
FcγR2A	21.95	13.48	300
FcγR2B	22.71	17.75	600
FcγR3A	23.80	5.50	300
FcRn	33.50		
B2M	11.41	4.10	300

Supplementary Figure S1: A) Detection of purified porcine FcγRs and FcRn (1 µg protein loaded per well) under non-reducing conditions in Coomassie blue-stained SDS-PAGE gels. **B)** Molecular weight based on amino acid sequence and the total protein yields for recombinant FcγRs in given expression scale.

Supplementary Figure S2

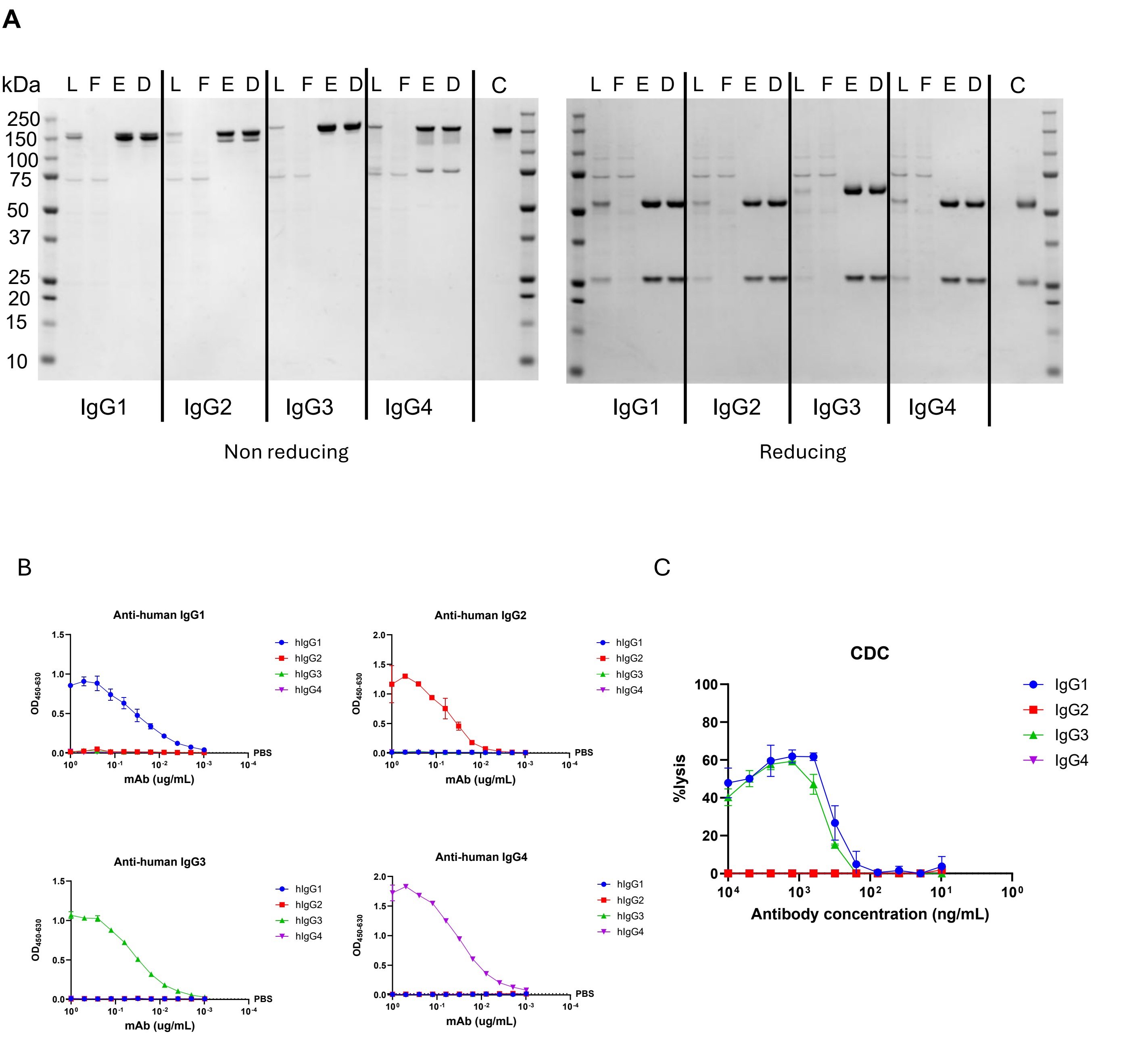

Supplementary Figure S2: **A**) SDS PAGE analysis of purified human 2-12C IgG1, IgG2, IgG3 and IgG4 subclasses in reducing and non reducing conditions (L: Load, F: Flow through, E: Elute and D: Dialysed fraction, C: IgG control). **B)** Detection of human IgG1, IgG2, IgG3 and IgG4 mAbs using commercially available anti human subclasses antibodies. **C)** CDC activity of IgG subclasses were measured on MDCK-HA incubated with a serial dilution of human IgG subclasses in the presence of rabbit complement.

Supplementary Figure S3

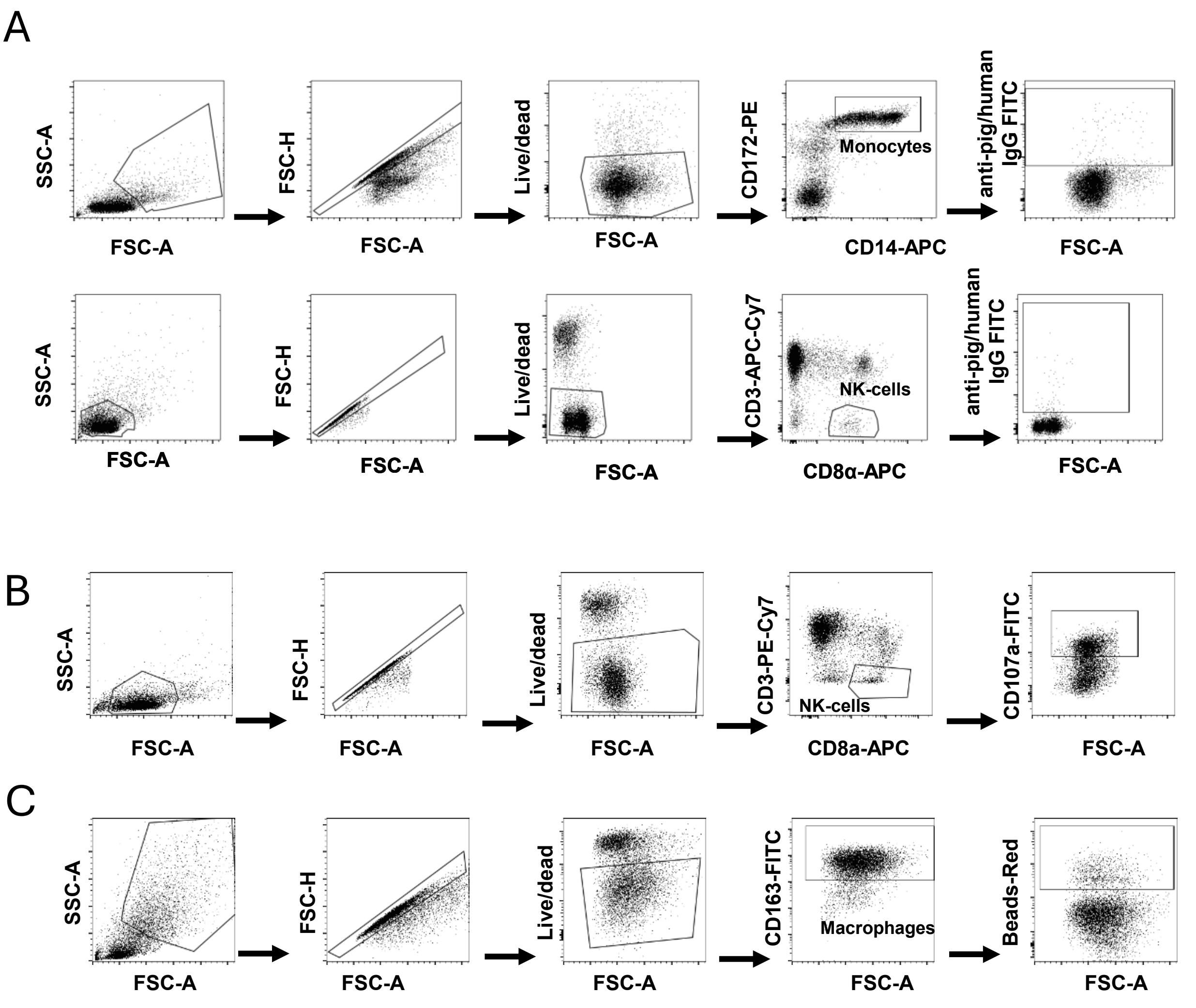

Supplementary Figure. S3. A) Gating strategy used for the identification of monocytes, macrophages, and NK cells. Sequential gating was applied to identify FSC/SSC-defined monocytes, lymphocytes, and macrophages, followed by singlet and live cells, $CD172a^{+}CD14^{+}$, monocytes, $CD3^{-}$ $CD8\alpha^{+}$ NK cells, and $CD163^{+}$ macrophages. **B)** Gating strategy for identification of NK cells expressing the degranulation marker CD107a within the $CD3^{-}$ $CD8\alpha^{+}$ population. **C)** Gating strategy for identification of BAL macrophages defined as $CD16^{+}$ cells phagocytosing fluorescent beads.

Supplementary Figure S4

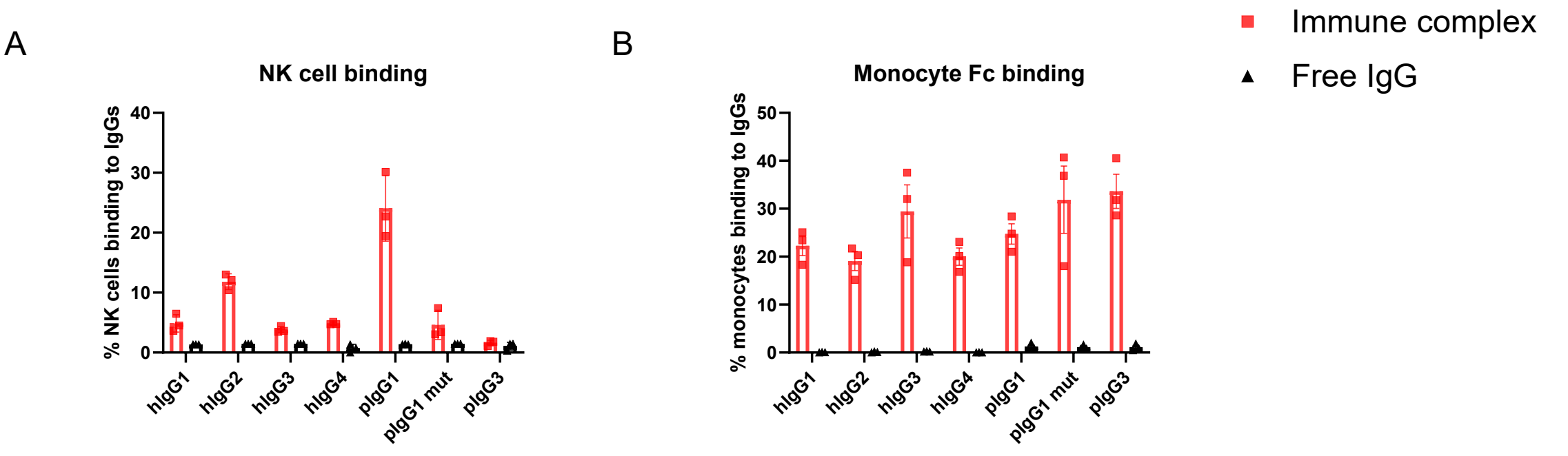

Figure 5. Binding of hIgG and pIgG subclasses to leucocytes. Binding profile of free (black triangle) and H1N1pdm09 virus immune complexed IgG subclasses (Red rectangle) to A) NK cells and B) monocytes. pIgG1 and pIgG3 were used as a control. Error bar indicates the mean and the standard deviation (SD), n=3.
